# Dimethyl itaconate alleviates the pyroptosis of macrophages through oxidative stress

**DOI:** 10.1186/s12865-021-00463-3

**Published:** 2021-11-08

**Authors:** Shan-Shan Huang, Dong-Yang Guo, Bing-Bing Jia, Guo-Long Cai, Jing Yan, Yan Lu, Zhou-Xin Yang

**Affiliations:** 1 The Second Clinical Medical Collage, Zhejiang Chinese Medicine University, Hangzhou, 310053 China; 2 Department of Critical Care Medicine, Zhejiang Hospital, 1229 Gudun Road, Hangzhou, 310030 China

**Keywords:** Dimethyl itaconate, Pyroptosis, Macrophages, Oxidative stress

## Abstract

Macrophages are involved in the pathophysiology of many diseases as critical cells of the innate immune system. Pyroptosis is a form of macrophage death that induces cytokinesis of phagocytic substances in the macrophages, thereby defending against infection. Dimethyl itaconate (DI) is an analog of itaconic acid with anti-inflammatory effects. However, the effect of dimethyl itaconate on macrophage pyroptosis has not been elucidated clearly. Thus, the present study aimed to analyze the effect of DI treatment on a macrophage pyroptosis model (Lipopolysaccharide, LPS + Adenosine Triphosphate, ATP). The results showed that 0.25 mM DI ameliorated macrophage pyroptosis and downregulated interleukin (IL)-1β expression. Then, real-time quantitative polymerase chain reaction (RT-qPCR) was used to confirm the result of RNA-sequencing of the upregulated oxidative stress-related genes (*Gclc* and *Gss*) and downregulated inflammation-related genes (*IL-12β* and *IL-1β*). In addition, Gene Ontology (GO) enrichment analysis showed that differential genes were associated with transcript levels and DNA replication. Kyoto encyclopedia of genes and genomes (KEGG) enrichment showed that signaling pathways, such as tumor necrosis factor (TNF), Jak, Toll-like receptor and IL-17, were altered after DI treatment. N-acetyl-L-cysteine (NAC) reversed the DI effect on the LPS + ATP-induced macrophage pyroptosis and upregulated the IL-1β expression. Oxidative stress-related protein Nrf2 is involved in the DI regulation of macrophage pyroptosis. Taken together, these findings suggested that DI alleviates the pyroptosis of macrophages through oxidative stress.

**Supplementary Information:**

The online version contains supplementary material available at 10.1186/s12865-021-00463-3.

## Background

Macrophages are essential cells of the innate immune system and play critical roles in the diseases such as sepsis [1]. Pyroptosis is cell death but distinct from apoptosis and necrosis. It is a general and natural immune effector mechanism, contributing to the inflammatory reaction in bacterial infections and various noninfectious diseases [2–4]. It is characterized by cell swelling, the formation of holes in the plasma membrane, and the release of pro-inflammatory cytokines, including interleukin (IL)-1β and IL-18. Thus, the process of pyroptosis exerts a dual effect: it protects the body from microbial infections and endogenous hazards, while excessive activation of pyroptosis leads to pathological inflammation [5]. Previous studies [6–8] have shown that macrophage pyroptosis is involved in the development of sepsis and that the regulation of the process pyroptosis may offer novel therapeutic approaches to sepsis.

Itaconic acid is a metabolite produced by the activation of immune cells, especially macrophages. The primary effect of the acid on the cellular metabolism during macrophage activation has been attributed to the inhibition of succinate dehydrogenase (SDH) [9, 10]. In addition, itaconic acid attenuates reperfusion injury by SDH and induces an antioxidant stress response [11]. It has a variety of anti-inflammatory, antioxidant, and immunomodulatory effects [12]. Itaconic acid and its membrane-permeable derivative, dimethyl itaconate (DI), selectively inhibit a subset of cytokines [9], including IL-6 and IL-12. A recent study showed that DI enhanced the survival rate, decreased the serum level of tumor necrosis factor-alpha (TNF-α) and IL-6, and ameliorated lung injury in septic mice. DI also suppressed the lipopolysaccharide (LPS)-induced production of TNF-α, IL-6, and nitric oxide synthase 2 in bone marrow-derived macrophages (BMDMs) [13].

Oxidative stress refers to the imbalance of oxidation and antioxidation in the body under the attack of harmful stimulating factors [14]. Moreover, cardiovascular, neurodegenerative, metabolic, and inflammatory diseases are known to be associated with oxidative stress [15], and the resulting reactive oxygen species (ROS) is considered to be the driving force of pyroptosis [16]. A study revealed that mitochondrial ROS promote macrophage pyroptosis by inducing gasdermin-D oxidation [17].

However, the role and mechanism of DI on macrophages pyroptosis have not yet been clarified. Therefore, in this study, the role and mechanism of DI on macrophage pyroptosis was analyzed by LPS + Adenosine Triphosphate (ATP)-induced pyroptosis model of BMDMs from C57BL/6 mice pretreated with DI.

## Methods

### BMDM isolation, culture, and treatment

Male C57BL/6 mice aged 6–8-weeks-old were purchased from Zhejiang Academy of Medical Sciences, Hangzhou, China. Following euthanasia by cervical dislocation, the lack of heartbeat was confirmed in each animal in accordance with the approved Zhejiang Academy of Medical Sciences protocol. BMDMs from the bilateral posterior femur of mice were rinsed using the DMEM (Genom, Hangzhou, China) medium. BMDMs were cultured in DMEM media supplemented with 50 ng/mL mouse recombinant macrophage colony-stimulating factor, 10% fetal bovine serum (FBS), penicillin (100 U/mL), and streptomycin (100 µM) in a humidified atmosphere containing 5% CO_2_ at 37 °C. After 7 days of culture, the cells were divided into different groups as follows: vehicle; DI (0.25 mM, Sigma, USA) + vehicle; Dimethyl sulfoxide (DMSO, Sigma, USA) + LPS (500 ng/mL, for 4 h, Sigma, USA) + ATP (5 mM, for 1 h, Sigma, USA); DI (0.25 mM, pre-treatment for 2 h) + LPS + ATP; N-acetyl-L-cysteine (NAC, 1 mM, Sigma, USA) or ML385(10 µM, Selleck, China), DI (0.25 mM, con-treatment for 2 h) + LPS + ATP. The concentrations were performed as described [18, 19]. The Ethics committee of the Zhejiang Academy of Medical Sciences approved the experimental protocol. All animal experiments met the ARRIVE guidelines [20].

### Cell viability assay

For cell viability assay, 5 × 10^3^ cells/well were seeded in 0.1 mL of DMEM supplemented with 10% FBS in a 96-well plate and cultured for 24 h, followed by treatment with a gradually increased concentration of DI (0.03125, 0.0625,0.125 and 0.25 mM) for 24 h. Then, 10 µL cell counting kit-8 (CCK-8,7Sea Pharmatech Co.Ltd., Shanghai, China) was added to each well and incubated at 37 °C for an additional 2 h. The absorbance was measured at 450 nm on a microplate reader (Thermo Scientific, San Jose, CA, USA).

### Propidium iodide (PI)-stained fluorescence microscopy

The cell mortality in each group was assessed via PI (BD Biosciences, USA) staining. The cells were incubated in a six-well plate at the density of 5 × 10^5^ cells/mL. The different groups were treated as described above and then incubated with 5 µL of PI for 10 min at room temperature in the dark. Subsequently, the cells were examined under an inverted fluorescence microscope (Nikon, Japan). Red presented PI-positive cells.

### Enzyme-linked immunosorbent assay (ELISA)

Cell-free supernatants were collected from each group and stored at – 80 °C. ELISA kits (Thermo Scientific, USA) for IL-1β was utilized following the manufacturer’s protocol.

### ROS detection

The level of ROS in DI + LPS + ATP group and NAC + DI + LPS + ATP group were detected by the dichlorodihydrofluorescein diacetate (DCFH-DA, Beyotime, China). Briefly, the cells were cultured in 96-well plates, treated as the previously described and incubated with 10 µM DCFH-DA for 30 min at 37 °C. After washing with DMEM thrice, the fluorescence intensity of ROS was detected with a fluorescence microplate reader (Thermo Scientific, San Jose, CA, USA) at 488 nm excitation wavelength and 520 nm emission wavelength. The concentration of ROS was expressed as fluorescence value.

### Real-time quantitative polymerase chain reaction (RT-qPCR) for mRNA expression

Total RNA was extracted from each group using the RNA Rapid Extraction Kit (Yishan, Biotechnology, Shanghai, China) and reverse transcribed into complementary DNA (cDNA) using the ReverTra-Ace-qPCR-RT kit (Toyobo Corporation, Osaka, Japan). Subsequently, qRT-PCR was performed using the SYBR green real-time PCR master mix (Toyobo) on a LightCycler 480 (Roche, Germany). *GAPDH* (glyceraldehyde-3-phosphate dehydrogenase) served as an internal control. The primers used are listed in Table 1.

**Table 1:** Target primer sequences

Target	Primer sequences
*Gclc* forward	5′-GGGGTGACGAGGTGGAGTA-3′
*Gclc* reverse	5′-GTTGGGGTTTGTCCTCTCCC-3′
*Ednrb* forward	5′-GAACAAGTGCATGCGAAACG-3′
*Ednrb* reverse	5′-ACTCAGCACAGTGATTCCCA-3′
*Gss* forward	5′-AGACCAAAGAAGCTTCCAAGAT-3′
*Gss* reverse	5′-ACCGCATTAGCTGAGCCATA-3′
*Acss2* forward	5′-GACCACCAAGATCACATACC-3′
*Acss2* reverse	5′-TTCTGAATGCCCTGTTTACG-3′
*Layn* forward	5′-CACATCACAGTTTAGGAACTGG-3′
*Layn* reverse	5′-GATGGCTGATGGTACATGAC-3′
*Edn1* forward	5′-TCTCTCTGCTGTTTGTGGCT-3′
*Edn1* reverse	5′-CCAGGTGGCAGAAGTAGACA-3′
*Fscn1* forward	5′-AACATCAAAGACTCCACGG-3′
*Fscn1* reverse	5′-AAGGAAGAAATCCACAGGG-3′
*IL-12β* forward	5′-GGAAGCACGGCAGCAGAATA-3′
*IL-12β* reverse	5′-AACTTGAGGGAGAAGTAGGAATGG-3′
*IL-1β* forward	5′-GCAACTGTTCCTGAACTCAACT-3′
*IL-1β* reverse	5′-ATCTTTTGGGGTCCGTCAACT-3′
*Saa3* forward	5′-TGCCATCATTCTTTGCATCTTGA-3′
*Saa3* reverse	5′-CCGTGAACTTCTGAACAGCCT-3′
*GAPDH* forward	5′-ATCAACGACCCCTTCATTGACC-3′
*GAPDH* reverse	5′-CCAGTAGACTCCACGACATACTCAGC-3′

### Library construction and sequencing

Total RNA was extracted from DMSO + LPS + ATP and DI + LPS + ATP groups using TRIzol (Thermo Fisher, USA). The experiments were performed in independent cultures from three mice. The mRNA was specifically captured using Dynabeads Oligo (dT) 25-61005 (Thermo Fisher, USA) and fragmented using NEBNext®UltraTM RNA Library Prep Kit for Illumina® (NEB, USA). cDNA was synthesized and constructed in the presence of reverse transcriptase (Invitrogen SuperScript™IIReverse Transcriptase, USA) library and sequenced. The processed clean data were aligned to the reference genome, and the expression was annotated and quantified using StringTie (2016) and gffcompare. Finally, gene expression obtained as fragments per kilobase of exon model per million mapped readsexon fragments (FPKM) was evaluated using ballgown.

### Analysis of differential transcripts

The differentially expressed mRNAs were selected with fold-change > 2 or fold-change < 0.5 and *P*-value < 0.05 using R package edgeR or DESeq2, followed by Gene Ontology (GO) enrichment and Kyoto Encyclopedia of Genes and Genomes (KEGG) enrichment analyses to identify the differentially expressed mRNAs.

### GO functional class and pathway enrichment analysis

The GO database reflects the distribution of the number of differentially significant genes on the GO term enriched in biological process, cellular component, and molecular function in the form of bar charts. KEGG is a database for the systematic analysis of correlations between genes and their coding products, gene function, and genomic information [21]. Also, pathways significantly enriched in expressed genes were identified.

#### Statistical analysis

Data were processed using GraphPad Prism version 7 and presented as mean ± standard deviation (SD), unless stated otherwise. The multigroup comparisons of means were carried out by one-way analysis of variance (ANOVA) test, with post hoc contrasts performed using Tukey’s multiple comparisons test. Paired t-test was used for comparison between groups. *P* < 0.05 indicated a statistically significant difference.

## Results

### Effect of DI on BMDM cell viability

**Fig. 1:**
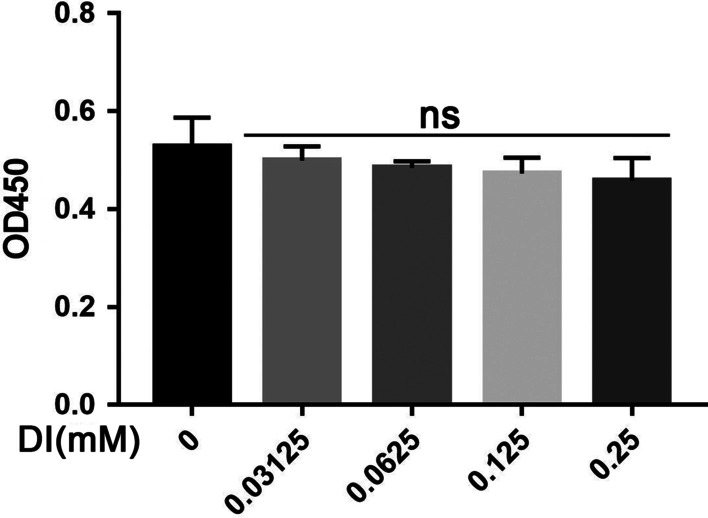
Effect of DI on BMDM cell viability. DI (0.03125, 0.0625, 0.125 and 0.25 mM) was added to cells, and the cell viability was detected after 24 h by CCK-8 *Vs.* Vehicle group; ns: not significant, *P* > 0.05)

The BMDM cells were treated with different concentrations of DI (0.03125, 0.0625, 0.125, and 0.25 mM) for 24 h. The CCK-8 assay showed that DI-treated groups did not differ in cell viability compared to the vehicle group (*P* > 0.05, Fig. 1).

### DI ameliorated cell mortality of BMDMs activated by LPS + ATP

**Fig. 2:**
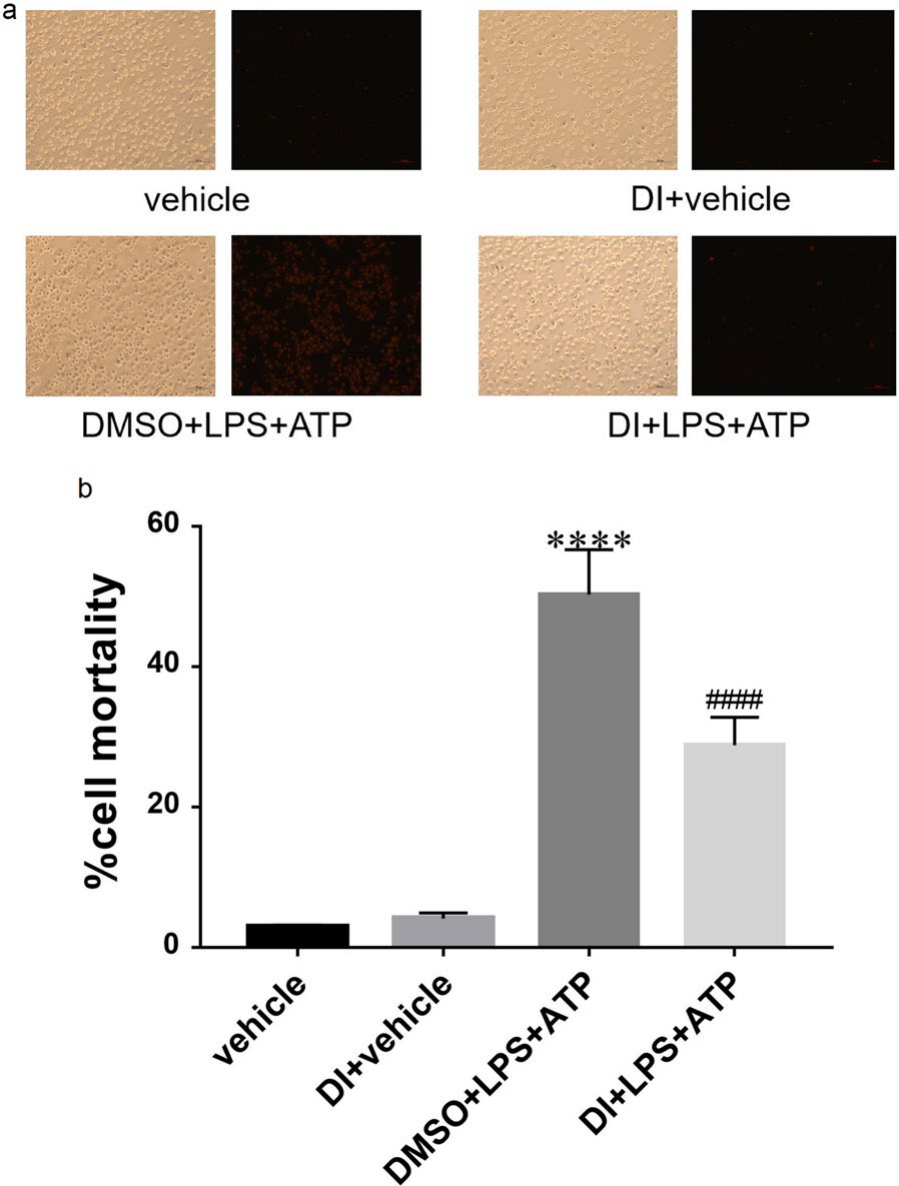
Effect of DI on cell mortality of BMDMs induced by LPS + ATP. **a** The cell mortality was observed by fluorescence microscopy after PI staining. **b** The cell number was quantified by counting in three random at 10×, and the mortality was expressed as mean ± standard error of the mean (SEM) (n = 3 per group, ^****^P < 0.0001 compared to the vehicle group; ^####^*P* < 0.0001 compared to the DMSO + LPS + ATP group)

The cell mortality of each group was detected by staining the cells with PI (Fig. 2a). As shown in Fig. 2b, the cell mortality of DMSO + LPS + ATP group was 50.27 ± 3.70% compared to the vehicle group was 2.97 ± 0.13%, indicating a significant increase (^****^P < 0.0001), while that of the DI treatment group was 28.80 ± 2.30% compared to the DMSO + LPS + ATP group, indicating a significant decrease (^####^*P* < 0.0001).

### DI decreased the level of IL-1β in BMDMs

**Fig. 3:**
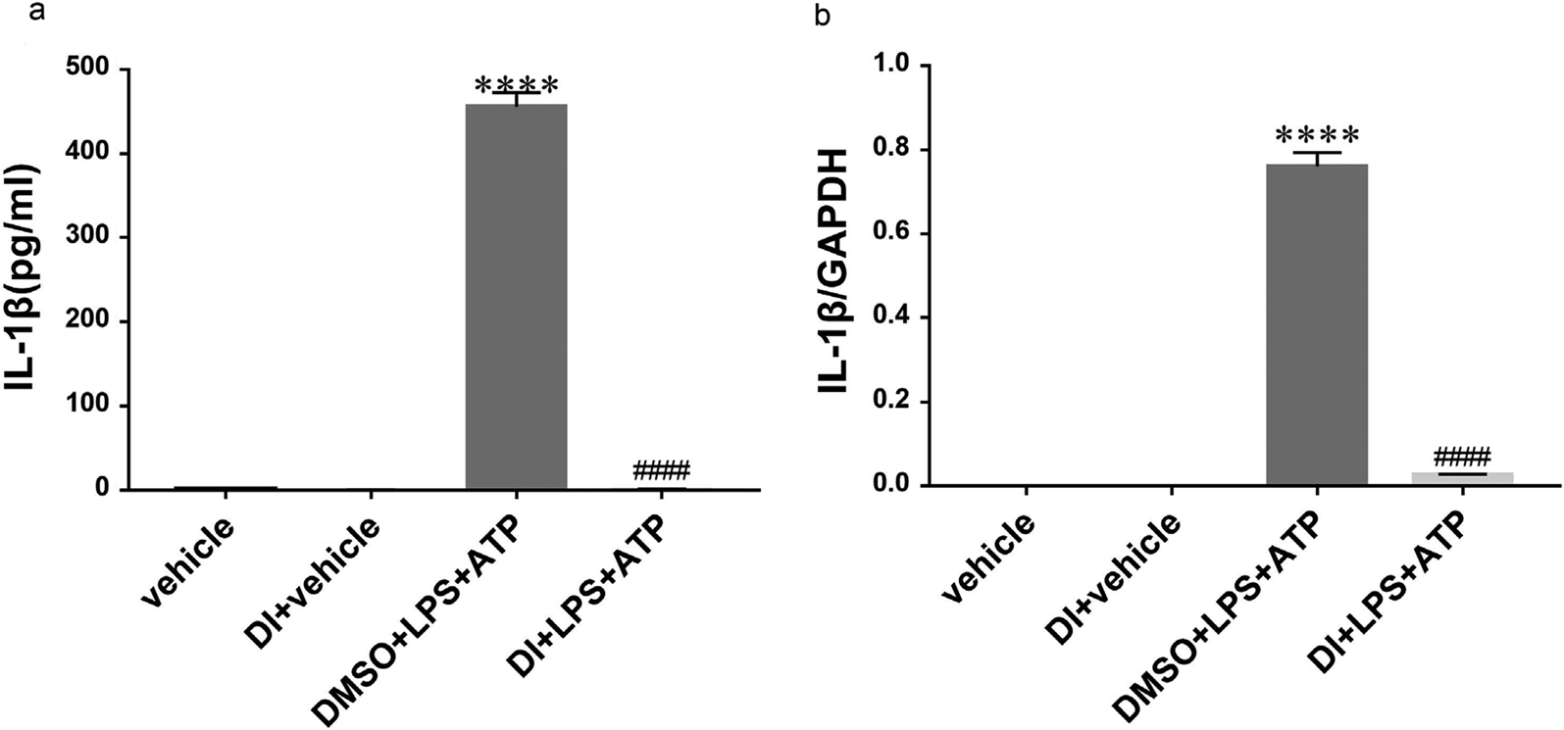
Effect of DI on IL-1β in BMDMs with LPS + ATP-induced pyroptosis. The concentration of IL-1β was detected by ELISA (**a**) and mRNA level was assessed by real-time PCR (**b**). (n = 3 per group, ^****^P < 0.0001 compared to the vehicle group; ^####^*P* < 0.0001 compared to the DMSO + LPS + ATP group)

The level of IL-1β in BMDMs was detected by ELISA and RT-qPCR. As shown in Fig. 3a, LPS + ATP-induced pyroptosis of BMDMs increased the serum levels of IL-1β, while DI treatment reduced the concentration of IL-1β. Similarly, DI treatment decreased the mRNA expression of *IL-1β* (Fig. 3b). These findings demonstrated that DI decreases the level of IL-1β in BMDMs.

### mRNA sequencing of DI treatment on the LPS + ATP induced pyroptosis in BMDMs

**Fig. 4:**
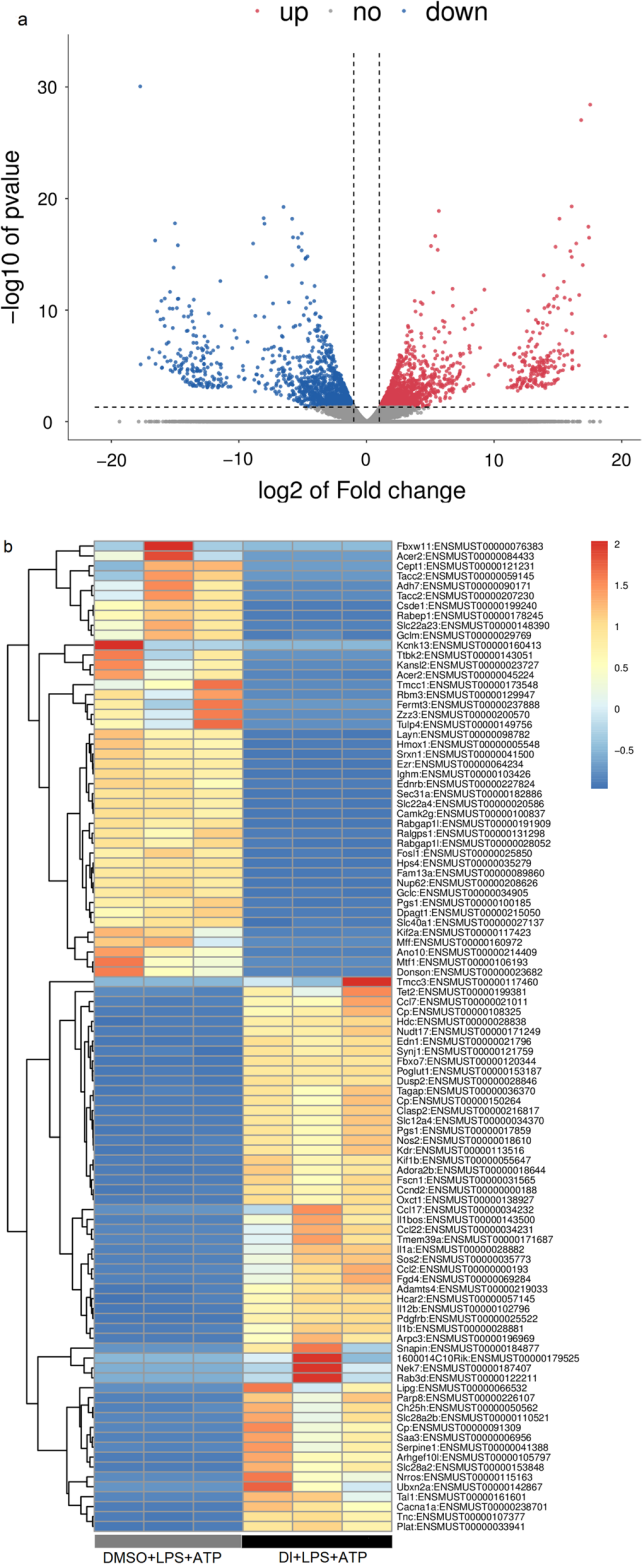

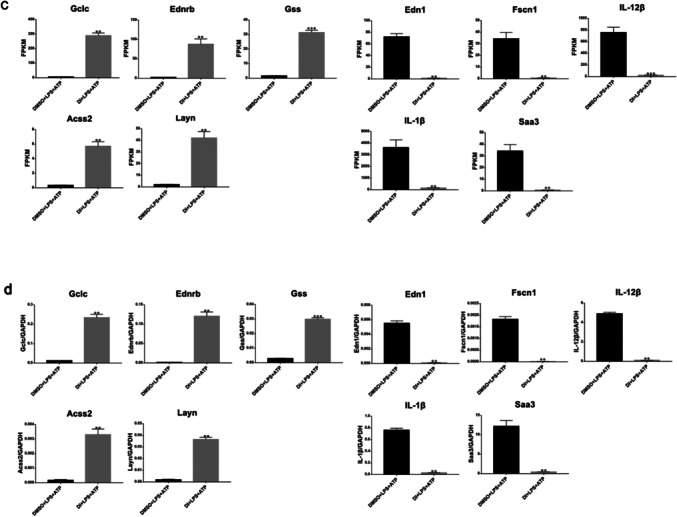
**a** Volcanic plots of sequencing results. **b** RNA-seq cluster analysis (abscissa: samples from different groups, ordinate: differential transcripts). **c** FPKM of sequencing results. d. Verification of mRNA expression of *Gclc, Ednrb, Gss, Acss2, Layn, Edn1, Fscn1, IL-12β, IL-1β,* and *Saa3* by real-time PCR (^**^P < 0.005, ^***^P < 0.001)

The comparative analysis of two groups was based on mRNA sequencing that identified 2040 differentially expressed genes (DEGs), including 983 upregulated and 1057 downregulated genes (Fig. 4a/b). The top five upregulated DEGs with the highest significance were *Gclc, Ednrb, Gss, Acss2, and Layn,* and the top five downregulated DEGs with the highest significance were *Edn1, Fscn1, IL-12β, IL-1β, and Saa3*. In Fig. 4c, FPKM was used from mRNA sequencing results (Additional file 1: Table S1). Real-time PCR was used to verify the expression of these genes (Fig. 4d), indicating that the expression trends of these ten genes were consistent with the sequencing results. All the data were statistically significant (^*^P < 0.05).

### GO enrichment of DEGs after DI treatment

**Fig. 5:**
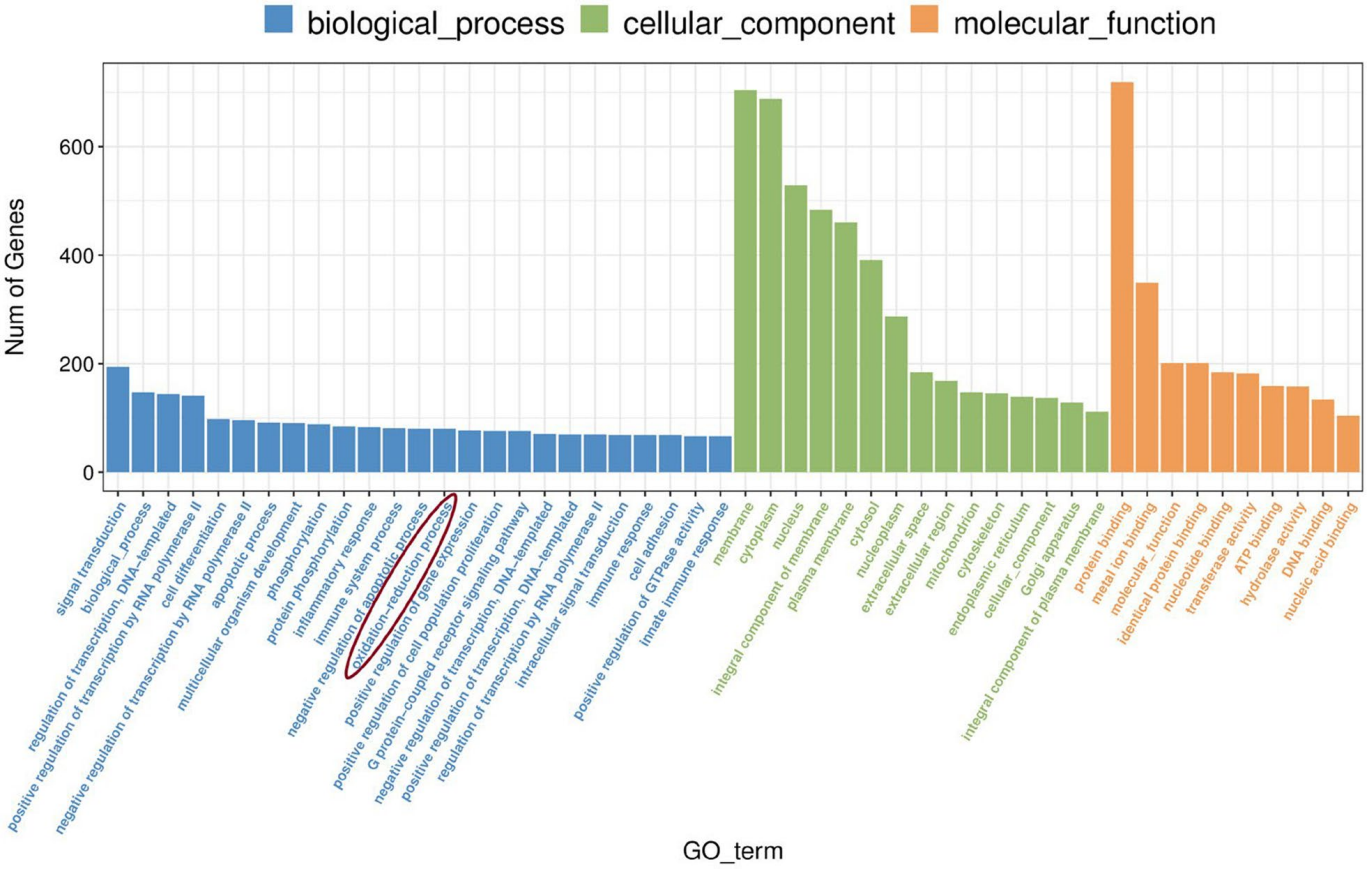
GO enrichment of genes after DI pre-treatment (abscissa: Number of genes, ordinate: GO-term)

In Fig. 5, the GO enrichment for the biological process of the enriched genes was signal transduction, biological process, regulation of transcription, DNA-templated, positive regulation of transcription by RNA polymerase II, and cell differentiation. Notably, DEGs were also enriched in the oxidation–reduction in the biological process. The GO enrichment for the cellular component of mainly enriched genes was membrane, cytoplasm, nucleus, an integral component of the membrane, and cytosol. The GO enrichment for molecular function of mainly enriched genes was protein binding, metal ion binding, molecular function, identical protein binding, and nucleotide-binding.

### KEGG enrichment of genes after DI treatment

**Fig. 6:**
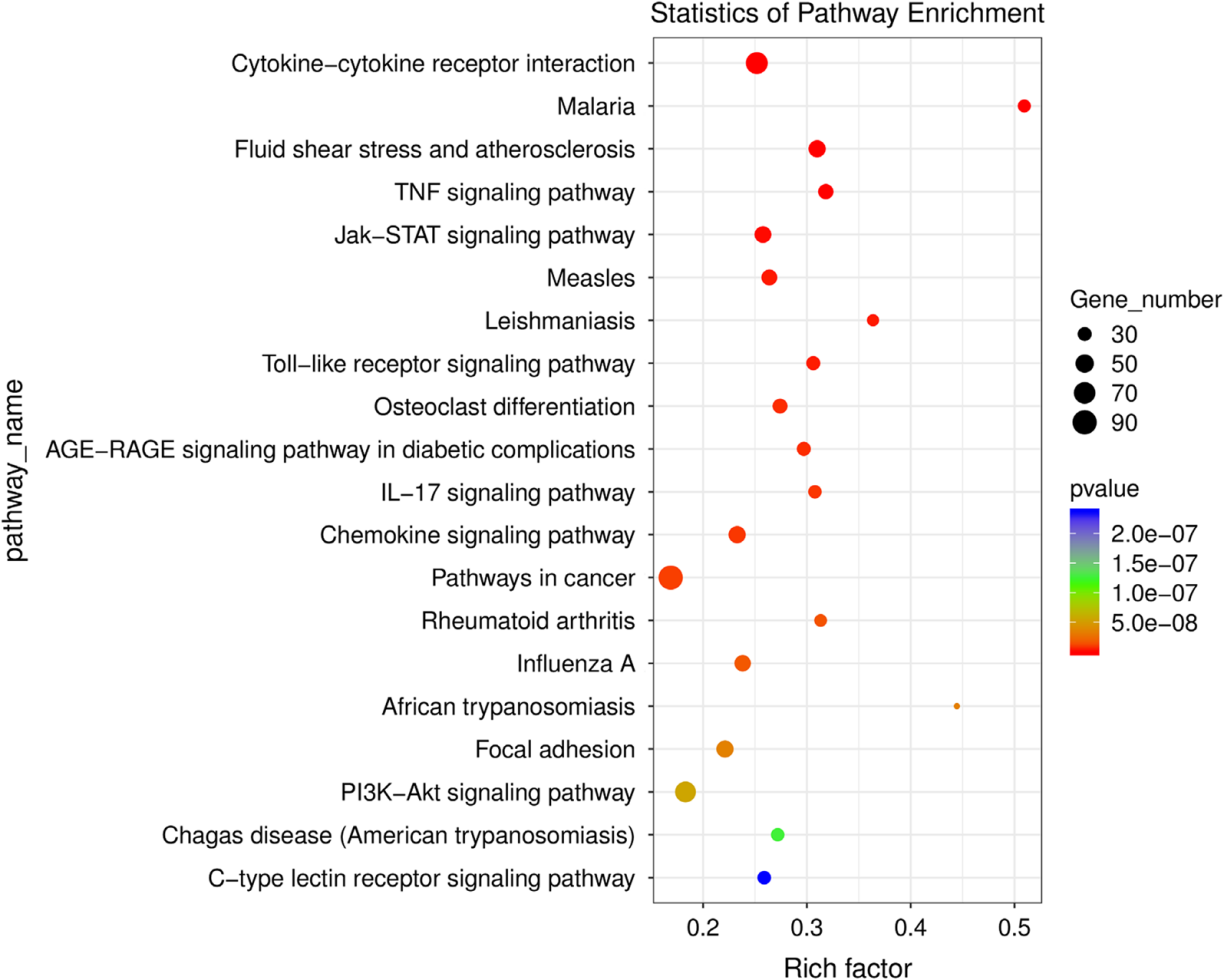
KEGG enrichment of genes after DI treatment (abscissa: pathway name, ordinate: rich factor, higher rich factor value, and pathway enrichment)

KEGG enrichment was used to explore the changes in the pathway after DI treatment. The KEGG enrichment of mainly enriched genes was cytokine-cytokine receptor interaction, malaria, fluid shear stress, and atherosclerosis, TNF signaling pathway, and Jak-STAT signaling pathway (Fig. 6). Thus, DI mainly affects the expression of inflammatory signaling pathways.

### NAC reversed DI effect on the LPS + ATP-induced pyroptosis of BMDMs

**Fig. 7:**
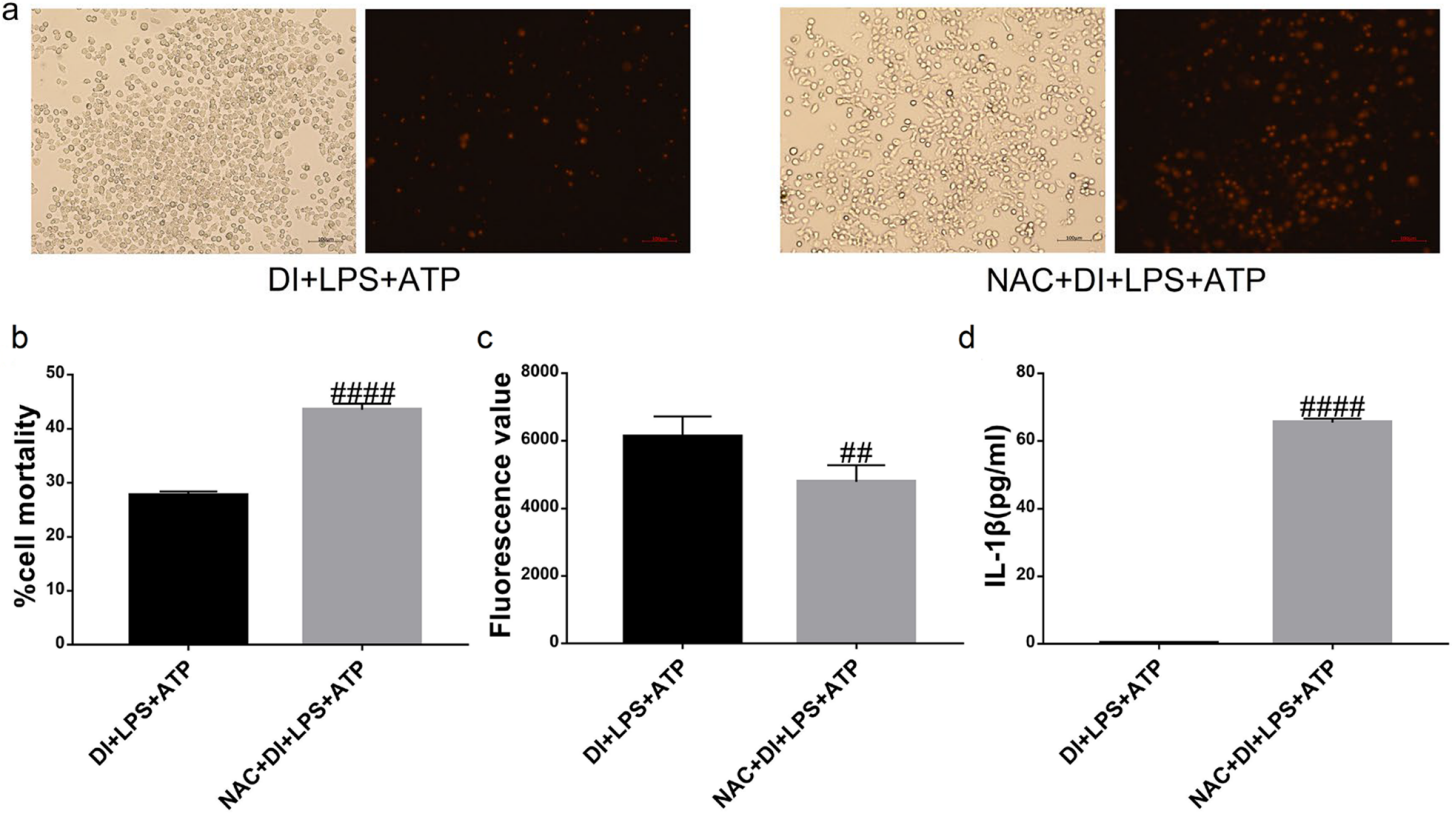
Effect of NAC on DI treatment of LPS + ATP-induced pyroptosis of BMDMs. **a** Cell mortality was observed by fluorescence microscopy after PI staining. **b** The cell number was quantified by counting in three random at 10×, and the mortality was expressed as mean ± SEM. **c** The concentrations of ROS were detected by the DCFH-DA and expressed as fluorescence value. **d** The concentration of IL-1β was detected by ELISA. (n = 3 per group, ^##^*P* < 0.005, ^####^*P* < 0.0001 compared to the DI + LPS + ATP group)

Based on the sequencing results, we found that DI significantly upregulated the oxidation–reduction-related genes (*Gclc* and *Gss*), and the differential genes in GO analysis were also enriched in the biological process of oxidation–reduction. Therefore, we speculated that the oxidation–reduction process plays an essential role in the effect of DI; hence, we treated DI-induced macrophage pyroptosis with NAC. Next, we detected cell mortality by staining cells with PI (Fig. 7a) and the level of ROS by the DCFH-DA in DI + LPS + ATP and NAC + DI + LPS + ATP groups (Fig. 7c). As shown in Fig. 7b, the cell mortality of the NAC + DI + LPS + ATP group (43.5 ± 0.64%) was significantly increased compared to that of the DI + LPS + ATP group (27.67 ± 0.41%), (^####^*P* < 0.0001). And the level of ROS in the NAC + DI + LPS + ATP group was significantly decreased compared to that of the DI + LPS + ATP group (^##^*P* < 0.005). Then, the expression of IL-1β in BMDMs was detected by ELISA (Fig. 7d). Compared to the DI + LPS + ATP group, the level of IL-1β in the NAC + DI + LPS + ATP group increased significantly. These findings proposed that NAC reversed the DI effect on the LPS + ATP-induced pyroptosis of BMDMs.

### ML 385 reversed DI effect on the LPS + ATP-induced pyroptosis of BMDMs

**Fig. 8:**
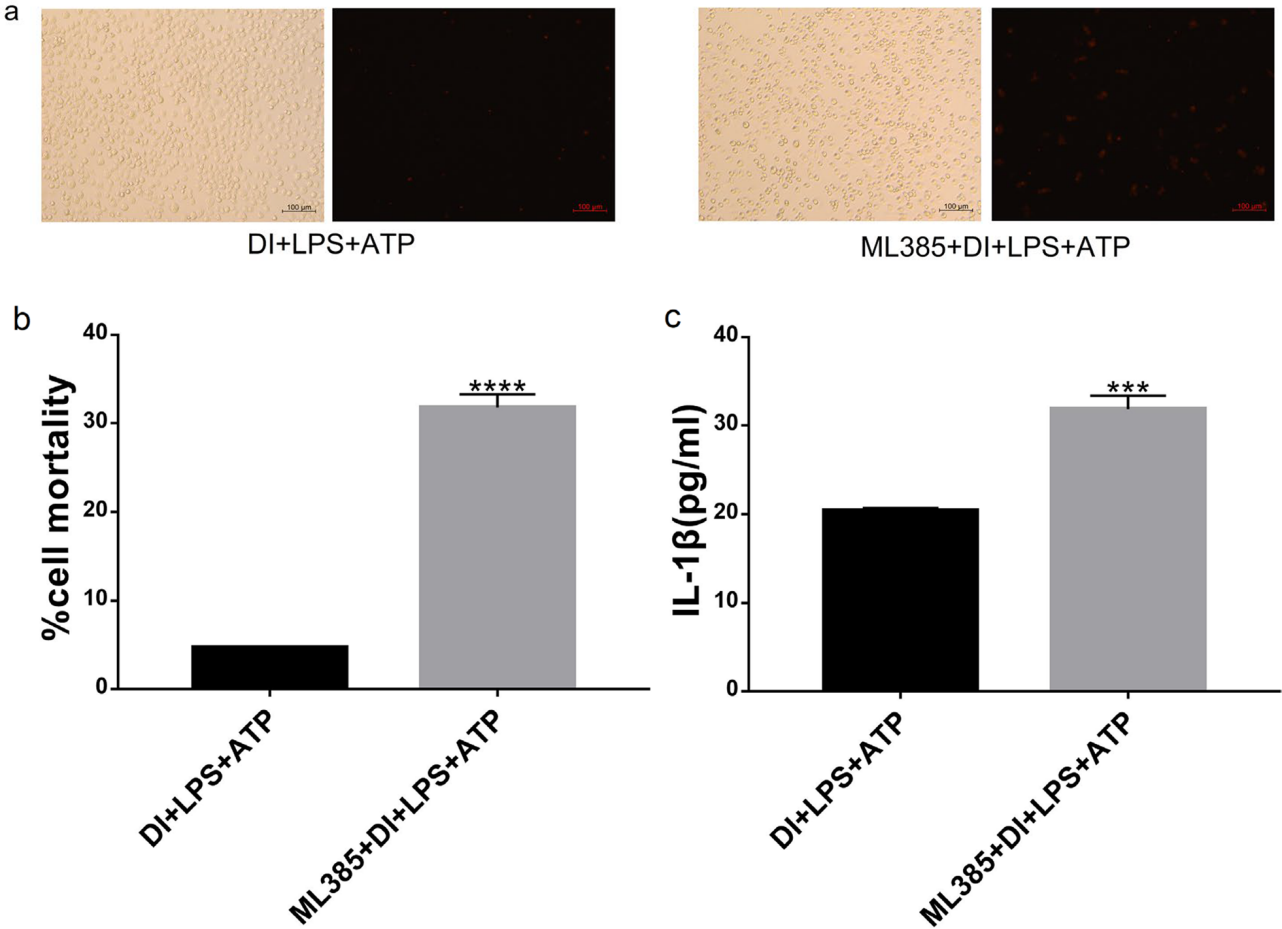
Effect of ML385 on DI treatment of LPS + ATP-induced pyroptosis of BMDMs. **a** Cell mortality was observed by fluorescence microscopy after PI staining. **b** The cell number was quantified by counting in three random at 10×, and the mortality was expressed as mean ± SEM. **c** The concentration of IL-1β was detected by ELISA. (n = 3 per group, ^***^P < 0.005, ^****^P < 0.0001 compared to the DI + LPS + ATP group)

Among upregulated DEGs with the highest significance, genes like *Gss, Gclc* and *Hmox1* [22, 23], were suggested to be regulated by the transcription factor NF-E2-related factor 2 (Nrf2), an important transcription factor that in response of the cellular oxidative stress. Thus, we co-treated macrophage pyroptosis model with ML385 (Nrf2 inhibitor) and DI. As shown in Fig. 8, ML385 similarly reversed the cell mortality (****P < 0.0001) and the level of IL-1β (***P < 0.0005) of DI effect on macrophage pyroptosis. In summary, these results indicated that oxidative stress-related protein Nrf2 is involved in the DI regulation of macrophage pyroptosis.

## Discussion

In the current study, we demonstrated that DI improves the cell mortality of LPS + ATP-induced macrophage pyroptosis and reduces the inflammatory factor IL-1β, while NAC could reverse this protective effect. We also used high-throughput sequencing to investigate the DI-treated macrophage pyroptosis and pyroptosis model and found that the upregulated differential genes (*Gclc* and *Gss*) were mainly associated with oxidation–reduction, while the downregulated differential genes (*IL-1β* and *IL-12β*) were associated with inflammatory responses. In the GO and KEGG enrichment analyses, we found that DI affects the biological process, including oxidation–reduction and the inflammatory signaling pathway. Besides, we also found that oxidative stress-related protein Nrf2 is involved in the DI regulation of macrophage pyroptosis. Thus, DI upregulated *Gss* and *Gclc* at the transcriptional level activating the antioxidant stress response and decreasing the level of IL-1β, thereby inhibiting macrophage pyroptosis.

Macrophage pyroptosis is involved in various inflammation-related diseases, such as psoriasis [24], osteoarthritis [25], and sepsis [8], and the associated inflammatory response can be attenuated by inhibiting macrophage pyroptosis. Some studies have shown that the regulation of macrophage pyroptosis mainly consists of Caspase-1-dependent classical pyroptosis pathways and non-Caspase-1-dependent non-classical pyroptosis pathways, which are complex signaling pathways. Moreover, the Keap1/Nrf2/HO-1 pathway can prevent pulmonary ischemia–reperfusion injury by reducing oxidative stress and promoting the antioxidant enzyme activity to inhibit alveolar macrophage pyroptosis [26]. In addition, the inhibition of the TNF-α/HMGB1 inflammatory signaling pathway suppresses macrophage pyroptosis to improve liver and kidney function during acute kidney injury and acute liver failure [27].

Macrophage pyroptosis is one of the ways of macrophage death, in addition to apoptosis and necrosis. A previous study showed that high dose itaconate surrogate 4-octyl itaconate (4-OI) induces apoptotic cell death independently of the classical inflammasome pathway [28]. Currently, there are no studies to confirm the association of DI with apoptosis and necrosis. It has been demonstrated that itaconic acid inhibits the activation of NLRP3 inflammasome in macrophage and reduces the level of IL-1β, which is negatively correlated with the level of intracellularly accumulated itaconic acid. DI is a cell-permeable itaconic acid analogue that is not metabolized to itaconic acid intracellularly, but has a strong electrophilicity and can downregulate the level of IL-1β [29]. The current study also confirmed that DI reduces IL-1β levels and alleviates cell death in macrophage pyroptosis. Nonetheless, additional studies are required to further investigate whether DI is associated with apoptosis and necrosis in the future.

Among the differentially upregulated genes, *Gclc* and *Gss,* were associated with redox response. Gclc and Gss are also key enzymes for glutathione synthesis (GSH). It has an antioxidant effect that maintains the stability of cell redox and avoids mass cell death [17]. Among the differentially downregulated genes, *IL-1β* and *IL-12β* are inflammatory factors. IL-1β is an upstream pro-inflammatory cytokine, and some studies reported that blocking IL-1β also reduces immunosuppression [30], which results in late sepsis. IL-12β is a cytokine of the IL-12 family and a key pro-inflammatory cytokine produced by macrophages [31]. Therefore, DI may further improve the inflammatory response to sepsis by increasing the level of anti-oxidative stress-related factors and decreasing the expression of inflammatory factors in macrophages at the transcriptional level.

In addition, differentially enriched genes in GO enrichment analysis of biological processes include oxidation–reduction process. Next, we compared cell deaths and IL-1β levels in the DI + LPS + ATP and NAC + DI + LPS + ATP groups by PI staining and ELISA and found that the number of cell deaths and the level of IL-1β was increased by NAC + DI co-treatment. NAC is an antioxidant, and in the presence of redox-active transition metals, it causes biological damage via thiol oxidation by the metal ion followed by the generation of superoxides, H_2_O_2_ and •OH. NAC exerts diverse, complex effects that are largely associated with maintaining the levels of intracellular glutathione (GSH) [32]. Besides, studies [22, 23] showed that Nrf2 could regulate genes, like *Gss, Gclc* and *Hmox1.* We used Nrf2 inhibitor and DI to co-treat the macrophage pyroptosis model, and found that it has a similar effect to NAC. In our study, we found that NAC reduced the level of ROS in the DI + LPS + ATP group and reversed DI effect on macrophage pyroptosis, and oxidative stress-related protein Nrf2 is involved in the DI regulation of macrophage pyroptosis.

Several studies have shown that DI modulates different signaling pathways to exert effects. DI protects against fungal keratitis by activating the Nrf2/HO-1 signaling pathway [33]. It also prevents LPS-induced mastitis by activating MAPK and Nrf2 and inhibits the NF-κB signaling pathway [34] and LPS-induced endometritis by suppressing the TLR4/NF-κB and activating the Nrf2/HO-1 signaling pathway [35]. The immunomodulatory effects of dimethyl chlortetracycline on IL-17-IκBς axis-induced inflammation were observed in an animal model of imiquimod-induced psoriasis [19]. In this study, KEGG enrichment analysis suggested a significant effect of DI on many signaling pathways such as TLR, IL-17, and PI3K-AKT, which are involved in the regulation of oxidative stress processes. Therefore, DI alleviates macrophage pyroptosis, the underlying mechanism is related to the oxidative stress response, but whether it is related to these signaling pathways needs to be investigated further.

## Conclusions

Taken together, DI alleviates the pyroptosis of macrophages through oxidative stress, which provides an experimental basis for the regulation of sepsis pyroptosis and a theoretical basis for anti-inflammation and suppression of oxidativess stress in clinical sepsis.

## Supplementary Information

**Additional file 1:** The ARRIVE Guidelines Checklist.

## Data Availability

The datasets generated and analysed during the current study are available in the GEO database, https://www.ncbi.nlm.nih.gov/geo/query/acc.cgi?acc=GSE185895 and the data presented in this study are available on request from the corresponding author.

## Abbreviations

DI: Dimethyl itaconate
BMDM: Bone marrow-derived macrophages
LPS: Lipopolysaccharide
ATP: Adenosine Triphosphate
NAC: N-acetyl-L-cysteine
DMSO: Dimethyl sulfoxide
CCK-8: Cell counting kit-8
KEGG: Kyoto Encyclopedia of Genes and Genomes
GO: Gene Ontology
ELISA: Enzyme-linked immunosorbent assay
PCR: Polymerase chain reaction
DNA: Deoxyribonucleic acid
SDH: Succinate dehydrogenase
GSH: Glutathione
GAPDH: Glyceraldehyde-3-phosphate dehydrogenase
Edn1: Endothelin 1
Fscn1: Fascin actin-bundling protein 1
IL-12β: Interleukin-12 beta
IL-1β: Interleukin-1 beta
Saa3: Serum amyloid A 3
Gclc: Glutamate-cysteine ligase catalytic subunit
Ednrb: Endothelin receptor type B
Gss: Glutathione synthetase
Acss2: Acetyl-CoA synthetase 2
Layn: Layilin
TNF: Tumor necrosis factor
Hmox1: Heme oxygenase 1
SD: Standard deviation
ROS: Reactive oxygen species
4-OI: 4-Octyl itaconate
Nrf2: NF-E2-related factor 2
DEGs: Differentially expressed genes
